# Quantifying Chemical Structure and Machine‐Learned Atomic Energies in Amorphous and Liquid Silicon

**DOI:** 10.1002/anie.201902625

**Published:** 2019-04-17

**Authors:** Noam Bernstein, Bishal Bhattarai, Gábor Csányi, David A. Drabold, Stephen R. Elliott, Volker L. Deringer

**Affiliations:** ^1^ Center for Materials Physics and Technology U.S. Naval Research Laboratory Washington DC 20375 USA; ^2^ Department of Physics and Astronomy Ohio University Athens OH 45701 USA; ^3^ Department of Engineering University of Cambridge Cambridge CB2 1PZ UK; ^4^ Department of Chemistry University of Cambridge Cambridge CB2 1EW UK

**Keywords:** amorphous materials, computational chemistry, continuous random networks, machine learning, silicon

## Abstract

Amorphous materials are being described by increasingly powerful computer simulations, but new approaches are still needed to fully understand their intricate atomic structures. Here, we show how machine‐learning‐based techniques can give new, quantitative chemical insight into the atomic‐scale structure of amorphous silicon (*a*‐Si). We combine a quantitative description of the nearest‐ and next‐nearest‐neighbor structure with a quantitative description of local stability. The analysis is applied to an ensemble of *a*‐Si networks in which we tailor the degree of ordering by varying the quench rates down to 10^10^ K s^−1^. Our approach associates coordination defects in *a*‐Si with distinct stability regions and it has also been applied to liquid Si, where it traces a clear‐cut transition in local energies during vitrification. The method is straightforward and inexpensive to apply, and therefore expected to have more general significance for developing a quantitative understanding of liquid and amorphous states of matter.

The structure of amorphous silicon (*a*‐Si) is widely approximated as a continuous random network with tetrahedral coordination,^1^ but its details are much more intricate: defective environments, such as threefold‐bonded “dangling bonds”, as well as the degree of medium‐range order, have been discussed.^2^ Together with experimental probes,^3^ atomistic computer simulations have been giving useful insight into *a*‐Si for decades,^4^ and large‐scale simulation models now contain up to hundreds of thousands of atoms.^5^ With the recent emergence of linear‐scaling machine‐learning(ML)‐based interatomic potentials reaching accuracy levels close to quantum mechanics,^6^ materials modeling is promising to become even more realistic—especially in describing amorphous solids,^7^ as recently shown for *a*‐Si.^8^

Still, there remains the more fundamental challenge of not only to describe amorphous structures but to truly understand them. Simple criteria are widely used, including atomic coordination numbers (here denoted as *N*) and bond angles, which both give information about short‐range order (SRO),^9^ or ring statistics as a representative for medium‐range order (MRO).^10^ However, we do not know of a previous simple and general numerical approach that can quantify SRO and MRO at the same time. And even more critically, these purely structurally‐based indicators cannot give information about the energetic stability of individual environments.

Here, we describe a general, ML‐based approach that quantifies local structures and local energies of all individual atoms in models of *a*‐Si. We first introduce a structural coordinate that unifies the description of SRO and MRO environments and then combine this structural information with a second, stability coordinate in a two‐dimensional plot. Both analyses rely on the “learning” of local structure, manifested in a mathematically well‐defined framework without parametric terms. The ability to “machine‐learn” local chemical knowledge is an emerging research theme throughout the discipline: ML‐predicted atomic energies have been used to understand the stability and chemical nature of molecules^11^ and crystal structures,^12^ and to accelerate structural optimization.^13^ Here, we transfer such analyses to the amorphous and liquid states, where there is an even more dire need for information about atomically resolved stabilities and properties.

Our object of study is an ensemble of *a*‐Si networks that we created in parallel ML‐driven molecular‐dynamics (MD) simulations: 512‐atom models of liquid Si were cooled to solidify into *a*‐Si (Figure 1 a).^8^ Slower cooling yields more ordered networks;^8^ hence, changing the cooling rate allows us to tailor the degree of order in the structures and to probe its influence on the properties. Remarkably, the most ordered structures we obtained (for quench rates of 10^11^ and 10^10^ K s^−1^), albeit still containing ≈1 % defects, are energetically more favorable by 0.02 eV/at. (at the DFT‐PBE level) than a fully tetrahedral‐like relaxed Wooten–Winer–Weaire (WWW) model,^1^ which is currently considered a gold‐standard model for *a*‐Si (see Supporting Information).

**Figure 1:**
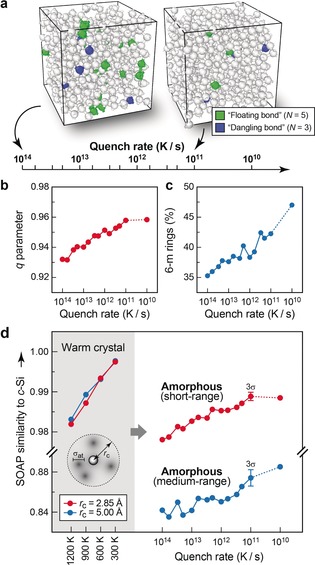
Progressively ordered *a*‐Si networks from melt–quench simulations with an ML‐based interatomic potential of quantum‐mechanical quality. a) Scale of cooling rates and associated required simulation times (1 ps requires 1000 MD time steps). Each tick corresponds to one independent MD simulation. Between 10^14^ and 10^11^ K s^−1^, we cooled at the respective constant rate; for the much more demanding 10^10^ K s^−1^ simulation, we varied the rate during the run (see Supporting Information). Two simulation cells are shown as examples and coordination defects are highlighted by coloring (green: over‐coordinated “floating‐bond” environments; blue: under‐coordinated “dangling‐bond” environments). b) Increasing short‐range order (SRO) in these systems, quantified using an established order parameter that returns unity for ideal tetrahedral environments.^9^ c) Increasing medium‐range order (MRO), assessed by counting 6‐membered rings.^10^ d) Unified description of both length scales using SOAP analysis. We first calibrated the SOAP kernel parameters (Table 1) for NNs (red) and NNNs (blue) using samples of thermalized *c*‐Si and then applied the method to our *a*‐Si networks. Median values over all atoms in the cells are given for each system. Error bars are shown for the SOAP values at 10^11^ K s^−1^ to estimate the scattering of the results; they indicate the threefold standard deviation for five additional, independent runs (see Supporting Information).

We start by illustrating the current state of the art. An established indicator for SRO in *a*‐Si measures how similar the atomic environments are to ideal tetrahedra, as probed via the bond angles.^9^ This order parameter increases as expected with slower quenching (increasing ordering) and then converges in a way that the median results for our 10^11^ and 10^10^ K s^−1^ structures are very similar (Figure 1 b). We also look at MRO via the count of six‐membered rings (Figure 1 c)—which is 100 % in diamond‐type *c*‐Si, where all atoms are in cyclohexane‐like rings, but smaller in *a*‐Si due to the presence of disorder. We stress again that these are two disjoint measures: bond angles cannot quantify MRO and the ring count does not give information about SRO.

To progress further, we now turn to the smooth overlap of atomic positions (SOAP) kernel,^14^ a mathematical approach that has been used with success to fit ML potentials^15^ and to analyze structures.^16^ In the SOAP formalism, the neighborhood density of the *i*‐th atom, smoothed by Gaussian functions with width *σ*_at_ and truncated by a cutoff function *f*_c_, is expanded into an atom‐centered basis of radial parts $R_{n}$ and spherical harmonics $Y_{lm}$ ,^14^

(1) $$\rho_{i}\left(r\right)=\sum_{j}\exp\left[-\frac{{\left(r-r_{ij}\right)}^{2}}{2\sigma_{at}^{2}}\right]f_{c}\left(r_{ij}\right)=\sum_{nlm}c_{nlm}^{\left(i\right)}R_{n}\left(r\right)Y_{lm}\left(\hat{r}\right)\;,$$

similar to how electronic wave functions are expanded in quantum chemistry. Based on the resulting combination coefficients, a similarity function or kernel can then be constructed, which provides a quantitative similarity measure on a scale from 0 to 1. However, the absolute value depends on the chosen cutoff radius and on the Gaussians that are placed on the atomic neighbors. To analyze both nearest‐neighbor (NN) and next‐nearest neighbor (NNN) environments, we here propose to combine two different SOAP kernels: that for the NN shell, making a sharp distinction between environments, and that for the NNN shell, being more tolerant to small structural changes. Therefore, we calibrate the fuzziness (via *σ*_at_) using *c*‐Si at *T*>0 K as a reference by requiring that the NN and NNN range of the SOAP values are similar in an ordered network with only thermal fluctuations (shaded area in Figure 1 d). We then apply the same pair of kernels to *a*‐Si: there, the increasing order with decreasing quench rate, the convergence of SRO, and the further increase of MRO are all correctly described within the same conceptual framework.

The simplicity and power of this NN–NNN kernel pair can also be shown by applying it to crystalline allotropes of Si (Table 1). In the lonsdaleite(hexagonal diamond)‐type form, the NN environments are as in the cubic diamond type (namely, ideal tetrahedra, with a similarity of 1.000), but the NNN environments differ, due to the rings being in boat rather than chair conformations, and hence the similarity to *c*‐Si drops to 0.974. We next look at an open‐framework Si allotrope, *oS*24, which was synthesized from Na_4_Si_24_ by sodium de‐intercalation.^18^ In *oS*24, the atoms are tetrahedrally coordinated, too, but with strong local distortions, and so the resulting NN values are comparable to those in *a*‐Si; the NNN values drop further, because the open framework structure is remarkably different from *c*‐Si. Finally, for β‐tin‐type Si with 4+2‐type coordination, the NN environments are clearly dissimilar to those in *c*‐Si, and the NNNs even more so (Table 1).

**Table 1:** SOAP parameters^14^ for the pair of kernels defined in this work and results for atomic sites in crystalline Si allotropes as obtained from both kernels.

			NN kernel	NNN kernel
Basis set size (*n*_max_, *l*_max_)			(16, 16)	(16, 16)
Cutoff radius *r*_cut_ [Å] Transition width *r*_Δ_ [Å]			2.85 0.30	5.00 0.60
Smoothness σ_at_ [Å]			0.30	0.60

Similarity of diamond‐type *c*‐Si and other allotropes				
	[lonsdaleite]	Si1≡Si2 (2*b*)	1.000	0.974
	*oS*24^17^	Si1 (8*f*) Si2 (8*f*) Si3 (8*f*)	0.980 0.994 0.987	0.792 0.908 0.747
	[β‐tin]	Si (4*a*)	0.723	0.342

We now consider the energies of the individual atoms, a crucial piece of information that cannot easily be obtained from DFT computations, which yield the total energy for the entire cell. In contrast, atomic energies are directly included in many ML‐based interatomic potentials by construction.^6^, ^18^ In the Gaussian approximation potential (GAP) framework we use here, the total energy is a sum of machine‐learned atomic energies that generally read^18^

(2) $$\epsilon_{i}=\sum_{j}\alpha_{j}K_{ij},$$

where the sum runs over a set of reference environments in the training database (index *j*) and two environments are compared using the similarity (kernel) function $K_{ij}$ , for which we use the SOAP formalism here. Hence, our structural analysis, GAP‐MD, and local energies all build on the same mathematical framework.

Figure 2 a shows that, indeed, machine‐learned atomic energies reveal the stability trends intuitively expected for *a*‐Si, the interpretation being qualitative for now. In the structural fragment shown in Figure 2 a, the dangling‐bond defect (*red*) has a high local energy, whereas the two tetrahedral‐like central atoms (white, blue) are more energetically favorable, depending on how strongly they are distorted. Histograms of these data, collected for a disordered, rapidly quenched structure (Figure 2 b) and a more ordered, slowly‐quenched structure (Figure 2 c) reveal that the energetic center of gravity for the more ordered *a*‐Si network does coincide with the experimentally determined stability.^19^ We note that such analyses are, in principle, possible with empirical interatomic potentials,^20^ but can be limited by the parametric shape of the potential. In contrast, our approach depends only on the input data, is readily generalized, and combines accurate DFT input data with a high‐fidelity ML fit whose uncertainty can be quantified15d to be in the meV range (insets of Figure 2 b, c).

**Figure 2:**
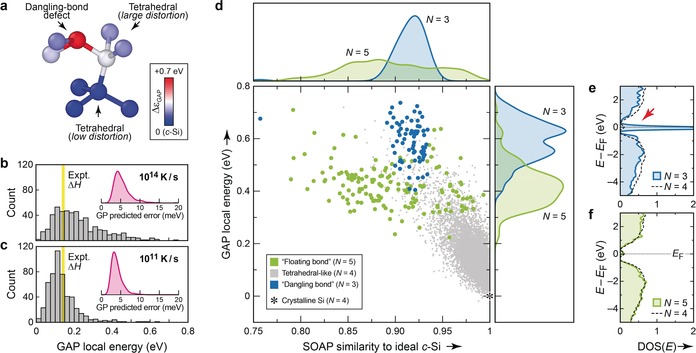
Machine‐learned atomic energies in *a*‐Si. a) Sample structural fragment, chosen to represent a dangling‐bond defect with high energy (red), a distorted tetrahedral environment with intermediate energy (white), and a more favorable tetrahedral environment with only low distortion (blue). Atoms are color‐coded according to their GAP atomic energy, *ϵ_i_*, given relative to that in diamond‐type *c*‐Si. b) Histogram of atomic energies in the structure quenched at 10^14^ K s^−1^. The experimental enthalpy of relaxed *a*‐Si (from Ref. 19, also relative to *c*‐Si) is indicated by yellow shading. The Gaussian‐process(GP)‐predicted error is indicated in the inset, showing a kernel‐density estimate for all atoms in the structure (see Supporting Information for details). c) Same analysis as in (b) for the more ordered 10^11^ K s^−1^ structure. d) 2D plot revealing the connection between structural order (NN similarity to diamond‐like *c*‐Si; horizontal axis) and GAP local energy for the individual atoms (vertical axis). Results are collected for all 14 systems, that is, for all quench rates from 10^14^ to 10^10^ K s^−1^ (see Figure 1). Kernel‐density estimates (smoothed histograms) are given for projections on both axes. e), f) Local electronic DOS for a structure quenched at 10^11^ K s^−1^ from Ref. 8, illustrating the very different electronic fingerprints of three‐ and fivefold‐bonded coordination defects. DOS plots are normalized per atom; for comparison, the average local DOS for all fourfold bonded atoms in the same structure is given by dashed lines. The red arrow in (e) highlights the mid‐gap states associated with dangling‐bond defects.

A 2D plot combining both quantities, the SOAP‐based diamond‐similarity and the local energy, is perhaps the most revealing form (Figure 2 d). Distinct energy regions at around +0.4 and +0.6 eV above that of ideal *c*‐Si are found for floating‐bond (*N*=5) and dangling‐bond (*N*=3) environments, respectively. The floating bonds show a wide structural variation within the NN shell, indicated by a large spread over the SOAP (*x*‐axis) coordinate, whereas dangling bonds clearly peak around a similarity value of 0.92. In this respect, the dangling‐bond atoms are clearly structurally closer to *c*‐Si than the 4+2‐type‐coordinated atoms in the β‐tin allotrope (Table 1), but they are considerably less diamond‐like than the median result for any of our *a*‐Si structures (Figure 1 d), which are dominated by fourfold‐bonded atoms. Looking at Figure 2 d again, it appears that the data points for dangling bonds (*N*=3) lie in the tail of a continuation of the plot for tetrahedral‐like environments (*N*=4); this is not the case for floating bonds (*N*=5). Finally, we note that the energies for *N*=4 atoms reach up to high values: their median is +0.14 eV, but the 98th percentile is at +0.42 eV and thus the remaining 2 % fourfold‐bonded atoms have energies that are higher than the median result for *N*=5 defects (+0.42 eV). This explains how our 10^10^ K s^−1^ structure, albeit having defects, can be lower in energy than the defect‐free WWW model.^1^

The higher GAP atomic energy (that is, larger instability) of dangling bonds compared to floating bonds is not only in line with previous theories,2a, 20 but it can also be confirmed by the electronic densities of states (DOS). For the energetically unfavorable dangling bonds (*N*=3), a large peak at the Fermi level, within the band gap, is seen from an atom‐resolved projection of the DOS (Figure 2 e). In contrast, the energetically more favorable floating bonds (*N*=5) make no pronounced mid‐gap contributions to the DOS (Figure 2 f).

This approach is expected to be general and to have wider significance. We show, as an example, an extension from the amorphous to the even more disordered liquid phase of silicon (Figure 3) which has been widely studied by simulations^22^ and experiments.^23^ Remarkably, the liquid appears to consist of atoms with a well‐defined normal distribution of GAP local energies, around +0.6 eV/at. above that of *c*‐Si. This distribution stays almost unchanged all the way until a narrow temperature window of approximately 1175 to 1195 K (Figure 3). There is a distinct transition with a bimodal distribution of atoms (center right panel in Figure 3) that our analysis suggests to be clearly either liquid‐like or amorphous‐like. Established empirical potentials^21^ fail to capture the nature of this transition, presumably because they have not been fitted to include the diverse structures in the liquid state. Further work will deal with detailed mechanistic studies of liquid Si using our new approach and with the extension to other systems: the ideas described here could be transferred to other tetrahedral networks such as the homologous material germanium or the highly complex amorphous forms of carbon7b (on which work is ongoing), or even to crystalline, amorphous, and liquid states of water.9b, 24

**Figure 3:**
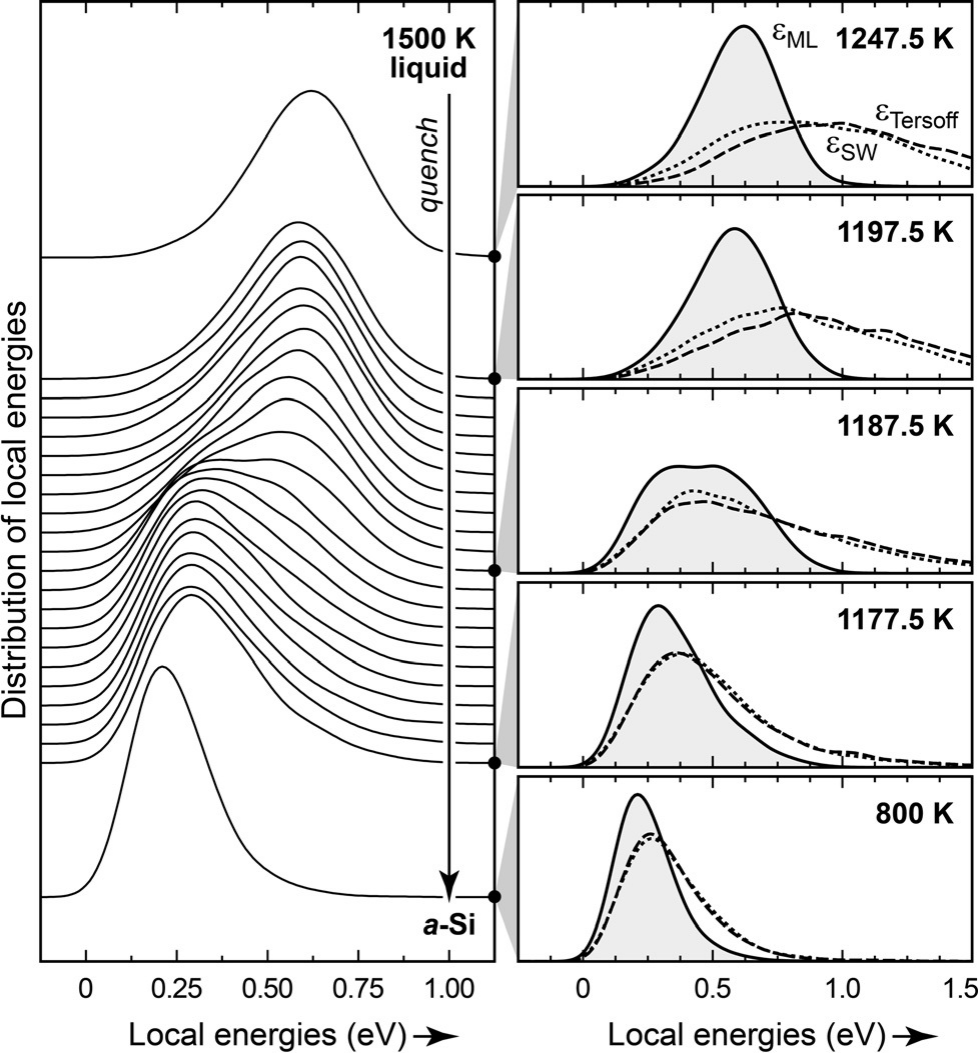
Machine‐learned atomic energies in liquid Si and their evolution during quenching into an amorphous configuration. Left: Kernel density estimates of local energies in a 4096‐atom system at selected points of an MD simulation (trajectory data from Ref. 8). Right: Close‐ups at given temperature values with results from two empirical potentials (Tersoff and Stillinger–Weber, SW; Ref. 21), overlaid for the same structures, evidencing the qualitatively different interpretation given by our approach.

## Conflict of interest

The authors declare no conflict of interest.

## Supporting information

As a service to our authors and readers, this journal provides supporting information supplied by the authors. Such materials are peer reviewed and may be re‐organized for online delivery, but are not copy‐edited or typeset. Technical support issues arising from supporting information (other than missing files) should be addressed to the authors.

Supplementary
