# Supplementary material for: Quantifying Chemical Structure and Machine‐Learned Atomic Energies in Amorphous and Liquid Silicon
Source: Angew Chem Int Ed Engl. 2019 Apr 17;58(21):7057–61. doi: 10.1002/anie.201902625 (PMC6563111; doi:10.1002/anie.201902625)
Supplement: Supplementary file 1 — Supplementary [file ANIE-58-7057-s001.pdf]

Angewandte Chemie
Eine Zeitschrift der Gesellschaft Deutscher Chemiker

Supporting Information

Quantifying Chemical Structure and Machine-Learned Atomic Energies in Amorphous and Liquid Silicon

*Noam Bernstein, Bishal Bhattarai, Gábor Csányi, David A. Drabold, Stephen R. Elliott, and Volker L. Deringer**

anie_201902625_sm_miscellaneous_information.pdf

Computational details

Generation of a-Si networks

Constant-pressure MD simulations (NPT ensemble), driven by a general-purpose GAP for Si (with the unique identifier `GAP_2017_6_17_60_4_3_56_165`),[S1] were carried out using LAMMPS.[S2–5] The system size was 512 atoms per cell, and the time step was 1 fs. After initial mixing at 1,800 K and keeping the system in the liquid state at 1,500 K, a quench to 500 K was performed with a given quench rate, and a final optimization was done using a conjugate-gradient (CG) minimizer. The computational protocol has been introduced and validated in a previous, more technical work;[S6] this includes comparison to the experimental structure factor (in particular, the inverse height of the first sharp diffraction peak) and to experimentally measured ^{29}Si NMR chemical shifts (see [S6] and references therein). Where appropriate, structural data were taken from our previous study (end points of 512-atom trajectories as well as selected intermediate points of a 4,096-atom trajectory resembling the liquid–amorphous transition),[S6] but used for new and different analyses (namely, SOAP structural similarity and GAP local energies) in the present work.

Furthermore, a new set of structures was generated to sample more finely spaced quench rates (*cf.* Figure 1a), adding data points at 6×10^{13} K/s, 3×10^{13} K/s, 2×10^{13} K/s, and so on. Besides "filling in" intermediate quench rates, we also expanded the scope of the simulations to a slowest rate of 10^{10} K/s, yielding a model with very high structural ordering (Figures 1b and 1c). A constant-rate quench at this rate would require 100 ns of simulation time (10^8 time steps, or several months of real time). We therefore use a variable-rate quenching scheme which was introduced and validated in [S6], in which the slowest quench rate (here, 10^{10} K/s) is only applied in the temperature region where it is found to be truly needed (viz. between 1,250 and 1,050 K), and a faster rate of 10^{13} K/s is applied elsewhere.

Local energies from GAP

Among the key findings of this work is that local energies in amorphous and liquid phases, as obtained from an ML-based interatomic potential by construction, permit chemical interpretation. We review the most important aspects here for convenience; the interested reader is referred to the original literature for a more detailed derivation.[S7–9] The local energy of the *i*-th atom, ε_i, is obtained in the Gaussian Approximation Potential (GAP) framework as follows:[S7]

$$\varepsilon_i = \sum_j \alpha_j K(\mathbf{q}_i, \mathbf{q}_j) \equiv \sum_j \alpha_j K_{ij}$$

(where the latter is just a shorthand notation). The sum runs over a set of reference configurations in the training database (index j), each described by a general descriptor vector denoted "$\mathbf{q}$", of which each is compared to the atom in question using the kernel function K. In the case of SOAP, this kernel is simply a properly normalized dot product, raised to a small integer power for better distinction between environments (here, $\zeta = 4$).[S8]

Depending on the physical nature of the system, more complex formulations may be used, but the general idea stays the same. For example, multiple descriptors (label d) can be combined by forming a linear combination with appropriate scaling factors $\delta^{(d)}$:[S9–11]

$$\varepsilon_i = \sum_d \delta^{(d)} \left(\sum_j \alpha_j^{(d)} K^{(d)} \left(\mathbf{q}_i^{(d)}, \mathbf{q}_j^{(d)} \right) \right).$$

Again, the result is one atomic energy value, ε_i. In the general-purpose silicon GAP we use, finally, the repulsive interaction between atoms at small distances is covered by a parametric two-body term ("core potential"), and the SOAP kernel is then used in the second term:[S1]

$$\varepsilon_i = \sum_j V_{ij}(r_{ij}) + \sum_j \alpha_j K(\mathbf{q}_i, \mathbf{q}_j).$$

For the sake of brevity, we have omitted the leading two-body potential term from the presentation in the main text. All these formulations lead to well-defined atomic energies ε_i, and we note that similar approaches can be followed with other ML-based interatomic potentials, such as artificial neural network potentials.[S12–14]

The full dataset of atomic energies (*cf.* Figure 2 in the main text) is provided in extended `XYZ` format, which makes it easily possible to identify the value corresponding to each atom (these files can be directly read into programs such as `ASE` or `quippy`) and as separate `csv` files. For convenience, in **Table S1**, we provide percentile values.

Table S1: Percentile values of the distribution of local energies for the 512-atom *a*-Si networks studied here (the 98-th percentile value means that 98 percent of the atoms have a local GAP energy below this value, *etc.*).

Percentile	N = 3 only	N = 4 only	N = 5 only	All atoms
100 **(Maximum)**	**0.737**	**0.720**	**0.710**	**0.737**
98	0.722	0.418	0.623	0.486
90	0.681	0.281	0.521	0.306
75	0.645	0.206	0.463	0.214
50 **(Median)**	**0.598**	**0.144**	**0.417**	**0.148**
25	0.544	0.101	0.364	0.103
10	0.494	0.070	0.332	0.071
2	0.427	0.037	0.283	0.038
0 **(Minimum)**	**0.405**	**–0.019**[a]	**0.224**	**–0.019**[a]

[a]The lowest value is slightly more negative than that of an atom in the optimized diamond structure, but the difference is within the maximum expected error (page S7–8).

Electronic structure

The local DOS plots shown in Figure 2e–f were obtained using an analytic projection scheme, as implemented in `LOBSTER` (www.cohp.de),[S15,16] ensuring a reliable transfer from a Γ-point VASP-PBE computation into a local basis of Slater-type orbitals (Si 3s 3p). The electronic wavefunction and the total DOS had already been computed in our previous work;[S6] here, this allows us to conveniently illustrate the effect of local environments. Further studies are currently underway to study more closely the electronic structure of the structural models generated here; these will be reported elsewhere.

Error bars for the SOAP similarity values

Figure 1d in the main text shows SOAP similarity values that compare NN and NNN atomic environments in *a*-Si to those in ideal *c*-Si. Due to the finite size of the structural models (512 atoms per cell), it is important to ask for the variability of the results presented; in other words, if a similar simulation is repeated for another 512-atom structure, how strongly will the SOAP results differ? To assess this question, we performed five independent quenching simulations, employing the variable-rate scheme discussed on page S1, with 10^{11} K s^{-1} in the temperature range from 1,250 to 1,050 K; 10^{13} K s^{-1} otherwise. For each system, we obtained the median NN and NNN similarity to *c*-Si (corresponding to the quantities plotted in Figure 1d) and calculated the standard deviation between the five systems (**Table S2**). In Figure 1d, we show error bars corresponding to three times this value ("3σ").

Table S2: SOAP similarity to *c*-Si (as in Figure 1d of the main text), but now for five *a*-Si structures that have been obtained in parallel quenching runs. Median values for the NN and NNN kernel are given for each cell, and below we list the averages and standard deviations over the five cells.

	NN median	NNN median
Run 1	0.986968	0.870881
Run 2	0.987224	0.869747
Run 3	0.987886	0.864289
Run 4	0.987775	0.872035
Run 5	0.987576	0.870733
Average over 5 runs	0.987486	0.869537
**Standard deviation**	**0.000343**	**0.002723**

Implementation and visualization

The local energies, including the predicted error, can be obtained with the freely available `QUIP/quippy` software (all required software, including the GAP prediction code, can be obtained at http://www.libatoms.org). The following `python` script yields this information:

Listing S1: A simple python script, illustrating how to use the GAP framework and `quippy` to predict local energies. As command-line arguments, this script takes (1) the coordinate file to be studied (extended `xyz` format), and (2) the GAP potential parameter file (`xml` format).

```
#!/Users/vld24/anaconda/bin/python2

from quippy import *
import sys

a = Atoms(sys.argv[1])

pot = Potential('IP GAP', param_filename=sys.argv[2])

a.set_calculator(pot)
a.set_cutoff(5.0)

a.get_potential_energies()

e = farray(0.0)
pot.calc(a, energy=e, args_str='local_gap_variance')

a.write('LOCAL_E_and_error_with_'+sys.argv[2]+'_'+sys.argv[1])
```

The information is included in a copy of the structure file, which is now amended by two additional columns in the extended `XYZ` format. The following is an example:

Listing S2: Example file header showing how the local energies and GAP variance are written by the script above. In the second line of the file, two new properties are listed (highlighted in green and cyan, respectively). For each atom (starting in the third line), these quantities are then given: note that for the local energy, these values are referenced to that of an atom in diamond-type *c*-Si (evaluated with the same GAP; ε(*c*-Si) = –163.17763895 eV), and that the errors are printed as variance (hence the square root must be taken to obtain a quantity in eV).

```
512

pbc="T T T" Lattice="21.90657164       0.00000000       0.00000000
   -0.01012491      21.96387922      0.00000000      0.20206325
  -0.08542605      21.79612182" Properties=species:S:1:pos:R:3:Z:I:
1:local_energy:R:1:local_gap_variance:R:1

Si   7.41226937   21.40807189   17.78143342   14     -163.08144625
0.00001970

Si   ...
```

Visualization was done using the freely available `OVITO` software (https://ovito.org/), which offers a convenient option to color-code atoms according to atomic properties.[S17]

Supplementary discussion (I): Characterization of *a*-Si networks

To further characterize the structural models that form the basis of this study, we provide key information about the most important ones in **Table S3** as well as additional raw data in **Table S4**. For this analysis, the structures were further relaxed using VASP.[S18,19] This changes the atomic positions only slightly: the GAP model used for melt–quenching is fitted to Perdew–Wang 91 (PW91) data, whereas the further relaxation here is done with the more widely used Perdew–Burke–Ernzerhof (PBE) functional[S20] for wider comparability. It does not affect the topological properties (connectivity, rings) of the networks.

The analysis presented here shows that our slowest-quenched structure outperforms the WWW model in terms of energies while having the same density and average coordination number. Furthermore, the simulated structure factor, $S(q)$, can be used as an additional measure for the quality of the structures because it can be compared to experimental data (**Figure S1**).

All structural models are provided as separate Supporting Information file (a `ZIP` archive containing structural data in extended `XYZ` and VASP `POSCAR` format).

Table S3: Information about relevant *a*-Si models as relaxed by DFT-PBE computations, including energies as given relative to the similarly relaxed WWW structure.

	ρ_0 (g cm^{-3})	**Coordination statistics** (%)			N_{avg}	ΔE (eV at.$^{-1}$)
		$N = 3$	$N = 4$	$N = 5$		
GAP (10^{14} K/s)	2.28	1.4	93.4	5.3	4.040	+0.087
GAP (10^{13} K/s)	2.26	1.0	96.5	2.5	4.016	+0.037
GAP (10^{12} K/s)	2.25	0.4	98.4	1.2	4.007	+0.008
GAP (10^{11} K/s)	2.26	0.6	98.4	1.0	4.004	**–0.022**
GAP (10^{10} K/s)	2.26	0.6	98.8	0.6	4.000	**–0.023**
WWW	2.24	–	100	–	4.000	0 (reference)

Table S4: Total DFT energies (raw values); median and maximum DFT force magnitude on atoms for the different models, showing that all structures are well relaxed.

	E (eV cell^{-1})	$\lvert\mathbf{F}\rvert_{med}$ (eV Å^{-1})	$\lvert\mathbf{F}\rvert_{max}$ (eV Å^{-1})
GAP (10^{14} K/s)	–2656.178043	0.002	0.019
GAP (10^{13} K/s)	–2681.359737	0.002	0.009
GAP (10^{12} K/s)	–2696.507605	0.003	0.010
GAP (10^{11} K/s)	–2711.581411	0.003	0.008
GAP (10^{10} K/s)	–2712.058318	0.002	0.007
WWW	–2700.479984	0.002	0.008

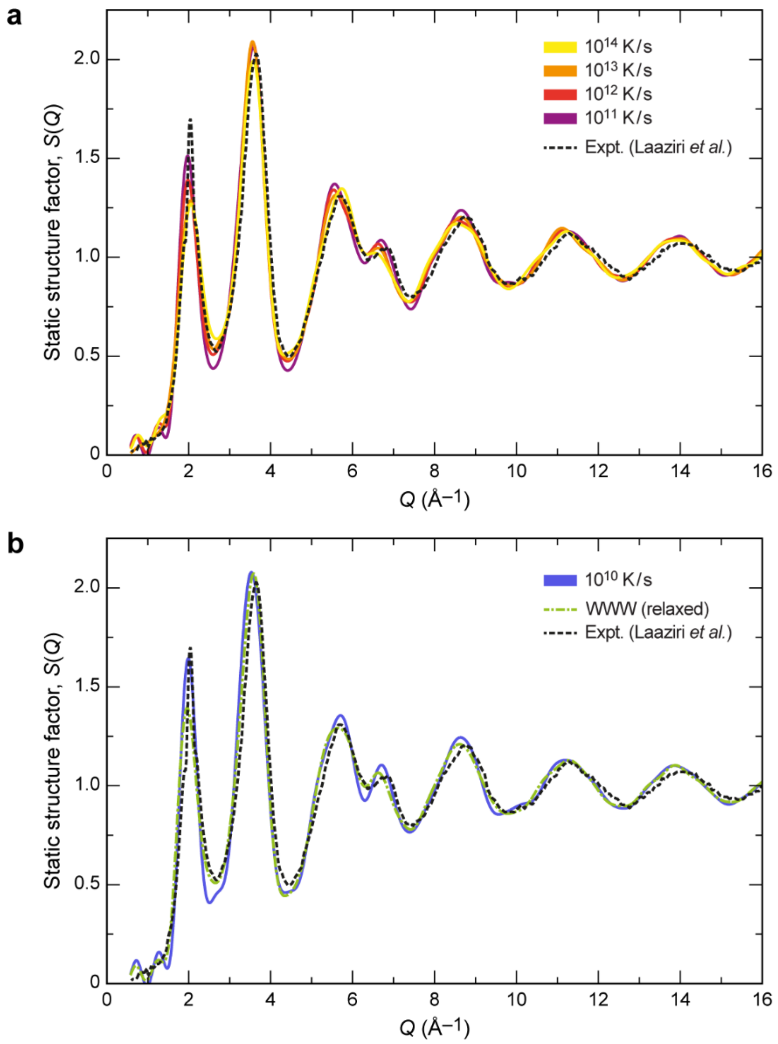

Figure S1: Static structure factors as a further means of validation for the different structure models. (a) Evolution of structure factors with decreasing quench rate, similar to [S6] (see there for a more detailed discussion on the link between structural order and features in the structure factors). Experimental data from Laaziri *et al.* (dashed black line; Ref. [S21]). (b) Structure factors for the newly generated 10^{10} K/s quenched structure (blue solid line) and the relaxed WWW network of similar size (dash-dotted green line), again showing experimental data from [S21] for comparison (dashed black line).

Supplementary discussion (II): Uncertainty quantification

The analysis of local energies can be further supported by the fact that Gaussian process regression makes it possible to quantify the expected error for any prediction. In other words, uncertainty quantification (an extremely important feature of ML models) is directly included in the GAP framework.[S1]

Recall that we obtain the atomic energy by summing up, over N reference configurations ($j = 1, \dots, N$), all pairs of fit coefficients α_j and kernel values K_{ij}:

$$\varepsilon_i = \sum_j \alpha_j K(\mathbf{q}_i, \mathbf{q}_j) \equiv \sum_j \alpha_j K_{ij} \, ,$$

This sum can be written as a dot product if we collect all entries for α_j and K_{ij} (for $j = 1, \dots, N$) into appropriate vectors, $\boldsymbol{\alpha}$ and $\mathbf{k}^{(i)}$:

$$\varepsilon_i = \boldsymbol{\alpha} \cdot \mathbf{k}^{(i)} \text{ with } \boldsymbol{\alpha} = \begin{pmatrix} \alpha_1 \\ \vdots \\ \alpha_N \end{pmatrix} \text{ and } \mathbf{k}^{(i)} = \begin{pmatrix} K_{i,1} \\ \vdots \\ K_{i,N} \end{pmatrix}.$$

We can then obtain the variance of the prediction (for the i-th atom) as follows:[S1]

$$\sigma_i{}^2 = K_{ii} - \left(\mathbf{k}^{(i)}\right)^{\mathrm{T}} (\mathbf{K} + \sigma_{\mathrm{e}}\mathbf{I})^{-1} \mathbf{k}^{(i)}$$

where σ_{e} is a small regularization value (on the order of 10^{-4} eV; [S1]). By taking its square root, we obtain an error measure with a dimension of energy:[S1]

$$\sigma_i = \sqrt{K_{ii} - (\mathbf{k}^{(i)})^{\mathrm{T}} (\mathbf{K} + \sigma_{\mathrm{e}}\mathbf{I})^{-1} \mathbf{k}^{(i)}}$$

We refer to this as a "Gaussian process (GP) predicted error", which we evaluated and plotted in **Figure S2** for the two structures characterized in Figures 3b (10^{14} K/s quench) and Figure 3c (10^{11} K/s quench), and show as a inset to Figures 2b–c in the main text. Additionally, values for the minimum, median, and maximum prediction errors are provided in **Table S5**.

Table S5: Predicted errors for GAP atomic energies (in eV per atom) for the two different structures characterized in Figures 2b–c.

	Fast-quenched a-Si (10^{14} K/s)	Slow-quenched a-Si (10^{11} K/s)
Maximum (σ_i)	0.0193	0.0109
Median (σ_i)	0.0048	0.0037
Minimum (σ_i)	0.0022	0.0018

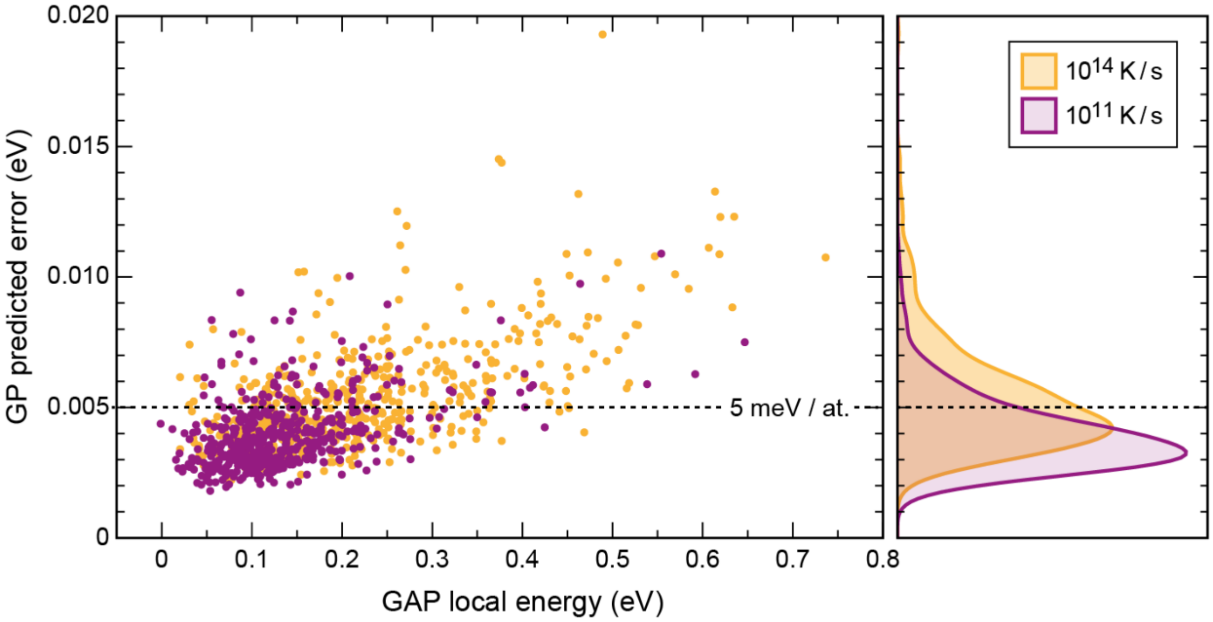

Figure S2: Uncertainty quantification by means of the predicted error for GAP local energies. The left-hand side shows all individual values plotted versus the corresponding atom's local energy; the right-hand side shows a kernel density estimate for a projection onto the *y*-axis. A dashed line indicates a value of 5 meV per atom that has been discussed in Ref. [S1] as a value indicating reliable predictions for various physical scenarios in crystalline silicon. The majority of data points is found below this value.

Supplementary references

[S1] A. P. Bartók, J. R. Kermode, N. Bernstein, G. Csányi, *Phys. Rev. X* **2018**, *8*, 041048.

[S2] S. Plimpton, *J. Comput. Phys.* **1995**, *117*, 1–19.

[S3] M. Parrinello, A. Rahman, *J. Appl. Phys.* **1981**, *52*, 7182–7190.

[S4] G. J. Martyna, D. J. Tobias, M. L. Klein, *J. Chem. Phys.* **1994**, *101*, 4177–4189.

[S5] W. Shinoda, M. Shiga, M. Mikami, *Phys. Rev. B* **2004**, *69*, 134103.

[S6] V. L. Deringer, N. Bernstein, A. P. Bartók, R. N. Kerber, M. J. Cliffe, L. E. Marbella, C. P. Grey, S. R. Elliott, G. Csányi, *J. Phys. Chem. Lett.* **2018**, *9*, 2879–2885.

[S7] A. P. Bartók, M. C. Payne, R. Kondor, G. Csányi, *Phys. Rev. Lett.* **2010**, *104*, 136403.

[S8] A. P. Bartók, R. Kondor, G. Csányi, *Phys. Rev. B* **2013**, *87*, 184115.

[S9] A. P. Bartók, G. Csányi, *Int. J. Quantum Chem.* **2015**, *115*, 1051–1057.

[S10] V. L. Deringer, G. Csányi, *Phys. Rev. B* **2017**, *95*, 094203.

[S11] S. Fujikake, V. L. Deringer, T. H. Lee, M. Krynski, S. R. Elliott, G. Csányi, *J. Chem. Phys.* **2018**, *148*, 241714.

[S12] J. Behler, *Angew. Chem. Int. Ed.* **2017**, *56*, 12828–12840.

[S13] N. Artrith, A. Urban, *Comput. Mater. Sci.* **2016**, *114*, 135–150.

[S14] E. L. Kolsbjerg, A. A. Peterson, B. Hammer, *Phys. Rev. B* **2018**, *97*, 195424.

[S15] S. Maintz, V. L. Deringer, A. L. Tchougréeff, R. Dronskowski, *J. Comput. Chem.* **2013**, *34*, 2557–2567.

[S16] S. Maintz, V. L. Deringer, A. L. Tchougréeff, R. Dronskowski, *J. Comput. Chem.* **2016**, *37*, 1030–1035.

[S17] A. Stukowski, *Model. Simul. Mater. Sci. Eng.* **2010**, *18*, 015012.

[S18] G. Kresse, J. Furthmüller, *Phys. Rev. B* **1996**, *54*, 11169–11186.

[S19] G. Kresse, D. Joubert, *Phys. Rev. B* **1999**, *59*, 1758–1775.

[S20] J. P. Perdew, K. Burke, M. Ernzerhof, *Phys. Rev. Lett.* **1996**, *77*, 3865–3868.

[S21] K. Laaziri, S. Kycia, S. Roorda, M. Chicoine, J. L. Robertson, J. Wang, S. C. Moss, *Phys. Rev. B* **1999**, *60*, 520–533.
